# Imported Ciprofloxacin-Resistant *Neisseria meningitidis*

**DOI:** 10.3201/eid1511.090833

**Published:** 2009-11

**Authors:** Giuseppe Lapadula, Franco Viganò, Paolo Fortuna, Alberto Dolara, Simone Bramati, Alessandro Soria, Sergio Foresti, Andrea Gori

**Affiliations:** San Gerardo Hospital, Monza, Italy

**Keywords:** Meningitis, Neisseria meningitidis, antimicrobial drug resistance, fluoroquinolones, ciprofloxacin, chemoprophylaxis, bacteria, letter

**To the Editor:** Emergence and spread of antimicrobial drug resistance in community-acquired infections is a global threat. Resistance of *Neisseria meningitidis* raises concern because of severity of disease caused by this organism and the need for immediate treatment of infected patients.

We report an imported case of meningococcal disease caused by fluoroquinolone-resistant *N*. *meningitidis*. The patient, a previously healthy, unvaccinated 43-year-old man who had traveled internationally, was hospitalized because of high fever, neck stiffness, and a diffuse petechial rash. Signs and symptoms were observed 24 hours after he had returned to Italy from a 10-day business trip during February–March 2009, to New Delhi and Chennai in India and a stopover of a few hours in Frankfurt, Germany.

Microscopic examination of cerebrospinal fluid showed gram-negative diplococci and culture documented *N*. *meningitidis* serogroup A. The strain was characterized as serotype 4,21 subtype P1.9 by using monoclonal antibodies. Multilocus sequence typing performed at the National Reference Laboratory for Invasive Meningococcal Diseases in Rome characterized the strain as sequence type (ST)-4789 and belonging to clonal complex ST-5/subgroup III.

Antimicrobial drug susceptibility was determined by using an agar dilution test, and MICs were determined by using an agar disk-diffusion test (Etest; AB Biodisk, Solna, Sweden) and standard techniques. The strain was resistant to ciprofloxacin, levofloxacin, and trimethoprim/sulfamethoxazole and susceptible to penicillin, ampicillin, ceftriaxone, chloramphenicol, rifampin, and azithromycin. MICs for ciprofloxacin, levofloxacin, penicillin, ampicillin, and ceftriaxone were 0.25, 0.25, 0.03, 0.12, and <0.016 mg/L, respectively (Figure). The patient recovered after treatment with ceftriaxone.

**Figure:**
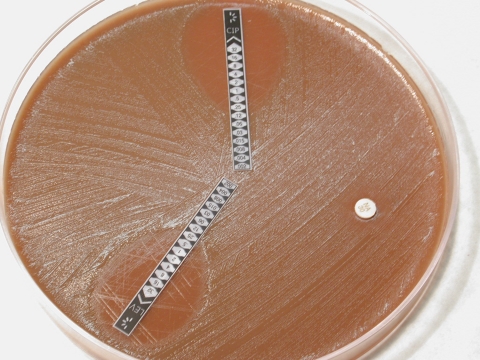
Antimicrobial drug–susceptibility test, showing resistance to levofloxacin (LEV, lower strip), ciprofloxacin (CIP, upper strip), and nalidixic acid (NA, disk) for the strain of *Neisseria meningitidis* isolated from the patient.

Before results of antimicrobial drug–susceptibility testing were available, 15 adult contacts of the patient received ciprofloxacin as chemoprophylaxis according to public health recommendations in Italy. After positive test results, all contacts were offered repeat chemoprophylaxis with rifampin; 13 of them accepted. A diagnosis of meningitis and results of antibiograms were sent to the patient’s place of employment in India and to the airport manager in Frankfurt. However, we were not able to assess what chemoprophylaxis was given to the patient’s fellow employees and air travel contacts. No secondary cases have been detected so far in Italy.

Sporadic cases of infection with *N*. *meningitidis* (mainly serogroup B) with reduced susceptibility to ciprofloxacin have been reported in Europe, North and South America, and Australia since 2000 (*1*–*4*). Ciprofloxacin-resistant *N*. *meningitidis* of serogroup A caused an outbreak of meningococcal meningitis in Delhi, India, in 2005 and a recurrence in 2006 (*5*). Although the patient reported in our study had no known contact in India with patients who had meningococcal disease, multilocus sequencing typing analysis showed that the isolate had the same sequence type as isolates from the epidemic in India (*5*,*6*).

We report isolation of an imported, ciprofloxacin-resistant strain of *N*. *meningitidis* isolated from a patient with meningococcal disease. During the past 2 years, 182 strains of *N*. *meningitidis* have been sent to the Istituto Superiore di Sanità; all were susceptible to ciprofloxacin and MICs ranged from 0.002 mg/L to 0.006 mg/L (National Reference Laboratory for Invasive Meningococcal Diseases, pers. comm.) Serogroup A *N*. *meningitidis* accounted for only 1 of these strains; serogroups B and C are the most common groups in Italy. In contrast, group A meningococci are the major cause of meningitis outbreaks worldwide, especially in Africa and Asia. To date, spread of ciprofloxacin resistance in serogroup A appears to be limited to India because a recent report of antimicrobial drug susceptibility of *N*. *meningitidis* in the meningitis belt of Africa during 2000–2006 showed no evidence of ciprofloxacin resistance (*7*).

Temporal correlation and epidemiologic features strongly suggest that transmission of *N*. *meningitidis* to our patient occurred during his journey to India. Meningococcal disease is rarely imported because onset of symptoms is often rapid and severe. Nonetheless, the enormous increase in global trade and travel and shortening of international travel time may increase the risk for spread of infectious diseases and drug-resistant organisms. In addition, carriage of *N*. *meningitidis* in the nasopharynx of otherwise healthy persons can occur.

Emergence of fluoroquinolone resistance in some countries raises concerns about current chemoprophylaxis recommendations for meningococcal disease. Ciprofloxacin is widely used for postexposure prophylaxis of close contacts of infected persons because it is simple to use (single oral dose) and lacks toxicity. However, patients and their contacts should be questioned about possible recent travel. When transmission of *N*. *meningitidis* is suspected in regions where fluoroquinolone resistance has been found (New Delhi, India, and North Dakota and western Minnesota in the United States), alternative chemoprophylaxis such as rifampin or ceftriaxone should be used.

Emergence of autochthonous ciprofloxacin-resistant *N*. *meningitidis* is possible in countries where fluoroquinolones are widely used. In vitro drug susceptibility testing is not routinely and uniformly used in all settings because treatment or chemoprophylaxis are usually started before antibiogram results are available. Our case demonstrates that drug susceptibility testing should be encouraged and routinely performed for all isolates. Local and worldwide surveillance for antimicrobial drug–resistant *N*. *meningitidis* is crucial for determining antimicrobial drug resistance trends and future recommendations for chemoprophylaxis and treatment.
